# Ameliorative effects of endogenous and exogenous indole-3-acetic acid on atrazine stressed paddy field cyanobacterial biofertilizer *Cylindrospermum stagnale*

**DOI:** 10.1038/s41598-022-15415-z

**Published:** 2022-07-01

**Authors:** Nazia Ahmad, Durdana Yasin, Fareha Bano, Tasneem Fatma

**Affiliations:** 1 Department of Biosciences, Jamia Millia Islamia, New Delhi, India; 2 Taibah University, Al Ula, Al Madinah Province Saudi Arabia

**Keywords:** Microbiology, Environmental sciences

## Abstract

Across the world, paddy fields naturally harbour cyanobacteria that function as biofertilizers and secrete various compounds like Indole-3-acetic acid (IAA) that help organisms in regulating their growth. Also, paddy field farming utilizes large amounts of pesticides (e.g. atrazine); but their continued application in the agricultural field causes toxicity in non-target cyanobacterial species that hinder their performance as a biofertilizer. Hence, the current study is an attempt to ameliorate the atrazine stress in cyanobacterium *Cylindrospermum stagnale* by addition of IAA (1 mM each) under different atrazine levels (0, 60, 80, 100, 120, 140 µg/l). Atrazine toxicity affected *C. stagnale* in a dose-dependent manner further experiments revealed that both the exogenous and endogenous IAA mitigated the detrimental effects of atrazine. It reduced MDA content and simultaneously increased chlorophyll content, total protein content, and multiple antioxidant enzyme activities [superoxide dismutase (SOD), catalase (CAT), and ascorbate peroxidase (APX)] at 140 µg/l. A molecular docking study revealed that the pesticide binds to the D1 protein of the photoelectric chain in photosynthesis. Hence, the application of IAA or cyanobacterial biofertilizer that secretes a sufficient amount of IAA may assist sustainable agriculture in counteracting the atrazine toxicity.

## Introduction

Pesticide toxicity affects humans due to their unrestricted use in agriculture. A lot of reasons, including the cheap price, uncomplicated accessibility, lack of awareness, and complete disregard for regulatory implementations have led to the uninterrupted application of pesticides^1^. Their long persistence in the environment leads to their bio-accumulation and bio-magnification ultimately leading to toxicity^2^.

Atrazine (1-Chloro-3-ethylamino-5-isopropylamino-2,4,6-triazine) is one such pesticides. It is a selective, most commonly used chlorinated triazine pesticide that exhibits acute and chronic phytotoxicity^3^. In agriculture, it is primarily utilized for controlling pre and post emergence of weeds in sugarcane, pineapple, sorghum, rice etc. and is sold under various commercial names like Gasaprim, Aatratol, etc. The high concentration of atrazine in agricultural field soil (0.002–0.015 mg/kg^4^ may represent a serious human health hazard because of the potential carcinogenic effects of triazines^5^. Due to its high mobility, it runoffs from these fields and also contaminates the water bodies in which its concentration may range from 0.1 to 180 μg/l^6^. As per World Health Organization (WHO), the concentration of atrazine should not exceed 2 μg/l^7^. In photosynthetic plants, atrazine has been shown to adversely affect the photosynthesis. In Soybean, it resulted in notable fluctuation of 66 abiotic-stress-related genes like wound-induced genes, universal stress-related genes and cold-stress-related genes and 12 Calvin cycle related genes like genes encoding RuBisCO activase, gene encoding carbonic anhydrase 2 and the ones encoding RuBisCO-associated proteins^8^. It upsets the growth of organisms by influencing photosynthesis causing the reactive oxygen species (ROS) generation^9^. At low concentrations, ROS are critical for cell signaling as they tweak the expression of genes and change specific defense proteins/enzymes activity. Interestingly, at increased concentrations, they result in oxidative stress. Inside the cell, antioxidant defense machinery (both enzymatic and non-enzymatic) manages the level of ROS under normal conditions^10^, whereas, under stress conditions, the cell demands additional support for the same. In such situations, it is compelling the scientist to find out strategies/strains resistant enough to tolerate the pesticides. One way of sustenance may be to use certain supplementations like plant hormones as growth enhancers.

Cyanobacteria are a group of prokaryotic organisms which are cosmopolitan in distribution. They are diazotrophic organisms which primarily contributes to the enhancement and maintenance of soil fertility^11^. Cellular adaptation of cyanobacteria to environmental stress is a major process that protects these organisms from deleterious effect of various stresses like salt, temperature, pesticide, heavy metals etc. Among many compounds that cyanobacteria produce, Indole-3-Acetic Acid (IAA) augments growth under normal conditions^12^ and also protects organisms under stress^13^. It is suggested that IAA enhances the antioxidant potential by changing the stress-responsive gene expressions^14^. It improves stress tolerance (to desiccation, osmotic, cold shock, heat shock, and oxidative) in *Triticum aestivum* and *Bradyrhizobium japonicum* by enhancing their antioxidant defense activities (catalase, superoxide dismutase, and peroxidase)^15,16^. It has also been shown to curtail the heavy metal (lead, zinc) toxicity on the shoot and root growth in *Helianthus annuus* L^17^ and nullify Furadan (pesticide) toxicity in *Nostoc* sp. and *Anabaena* sp.^18^. To the extent of our awareness, no other research work has been done to determine the effects of IAA on atrazine-stressed organisms. However, in other stresses like salt stress and heavy metals, few studies have been done. IAA considerably mitigates the adverse effects of salt in *Phaseolus mungo* L. wheat plants due to enhanced photosynthetic pigment concentration, impaired membrane permeability, and modified antioxidant enzyme activities^19,20^.

The research work aimed to check the potential of IAA (both exogenous and endogenous) in ameliorating commercial-grade atrazine toxicity in non-target nitrogen-fixing cyanobacterial strain *Cylindrospermum stagnale*.

## Material and methods

### Test strains and growth conditions

The test strain (*C. stagnale*) was procured from IARI and grown in a BG-11 medium (pH 7.3) under axenic conditions^21^. Experimental cultures were incubated in identical conditions (25 µmol photon m^−2^ s^−1^ light intensity,12:12 h light–dark photoperiod; 30 ± 1 °C temperature and 7.4 pH) for 24 days.

For evaluating atrazine toxicity, the pattern of progressive growth of test strain was measured as absorbance at 560 nm every third day for 24 days in the presence of 0, 60, 80, 100, 120, and 140 µg/l of atrazine (10% E.C.) as decided after calculating the LC_50_ of atrazine against the studied cyanobacterium. The atrazine was dissolved in DMSO and IAA was dissolved in ethanol with their final concentration in the media being > 3 μl/l and > 3 ml/l respectively. The biomass was filter harvested and air-dried overnight in the laminar hood. The experiments were carried out in triplicates.

### Chemicals

Analytical grade chemicals were purchased from Merck India Pvt. Ltd., and Hi-Media Laboratories Pvt. Ltd., Mumbai (India). Commercial grade atrazine was obtained from the Institute of Pesticide Formulation Technology, Gurugram (India).

### Growth of *C. stagnale* under atrazine stress

To determine the effect of atrazine on cyanobacterial growth, the LC_50_ value of atrazine for *C. stagnale* was determined in terms of quantitative estimation of dry biomass weight of the atrazine treated *C. stagnale*, and accordingly, various concentrations of the atrazine were used in all further experiments.

The pattern of progressive growth of *C. stagnale* was evaluated by estimating the light scattering of cell suspension at 750 nm, cell dry weight, and chlorophyll-'a' content every third day for 24 days. All the experiments were designed in triplicate.

Cell dry weight measurements were recorded from 0 to 24 days to ascertain progressive growth. For this, algal biomass was scrapped from the walls and bottom of the flask with a spatula and centrifuged at 6000×*g* for 10 min. For cell dry weight measurements cyanobacterial pellets were blotted dry on Whatman filter paper (No. 1) and oven-dried at 80 °C to a constant weight.

Chlorophyll-'a' content was evaluated by following the protocol developed by Mackinney^22^. Briefly, 10 mg cell biomass was extracted with 10 ml 95% (v/v) methanol for half an hour at 65 °C. The obtained extract was brought to the 25 °C and the supernatant obtained after centrifugation at 10,000×*g* for 10 min at 4 °C was used for chlorophyll-'a’ content estimation by calculating the absorbance of the methanol extract at 650 and 665 nm (Blank: methanol 95%) using the equation:

1 $${\text{Chlorophyll - a }}\left( {{\text{mg}}/{\text{g}}} \right) \, = { 2}.{55 }\left( {\lambda_{{{65}0}} \cdot { 1}0^{{ - {2}}} } \right) \, + \, 0.{4 }\left( {\lambda_{{{665}}} \cdot { 1}0^{{ - {2}}} } \right)$$

### Specific growth rate (µ) of *C. stagnale*

The specific growth rate is the derivative of population w.r.t. time overpopulation and was estimated according to the equation

2 $$\mu = \frac{1}{N}\frac{dN}{{dt}} = \frac{d\ln N}{{dt}}$$

where µ, N, and t denote specific growth rate, population, and time, respectively.

### Effect of IAA on biochemical parameters under atrazine stress

For biochemical analysis, the test cyanobacterial strain was grown under four different conditions in duplicate. In the first set, pure culture was present and it acted as control; in the second set, atrazine was added to the culture to see its effect on the cyanobacterium; in the third set, l-tryptophan (1 mM) was added to the culture containing atrazine for determining the influence of endogenous IAA (as l-tryptophan act as IAA precursor); and in the fourth set, IAA (1 mM) was added to the culture containing atrazine to analyse the impact of exogenous IAA.

Cultures were incubated for 24 days as discussed earlier. After 24 days, biomass was filter harvested for estimation of photosynthetic pigment (chlorophyll) content, total protein content, malondialdehyde (MDA) content, and superoxide dismutase (SOD) activity, ascorbate peroxidase (APX) activity, and catalase (CAT) activity.

### Estimation of cellular constituents (chlorophyll and proteins)

Chlorophyll was estimated by adopting the protocol given by Mackinney^22^ as mentioned earlier.

For estimation of protein in the test cyanobacterium, the protocol by Lowry et al.^23^ was followed, and BSA was taken as the standard and expressed as mg/g of the dry weight of the cultures.

### Estimation of lipid peroxidation as MDA

Lipid peroxidation as malondialdehyde (MDA) was estimated by following the protocol of Heath and Packer^24^. Briefly, cyanobacterial biomass (50 mg) was dissolved in trichloroacetic acid (1.5 ml,1%) and centrifuged at 10,000×*g* for 5 min at 4 °C. 1 ml of the supernatant was mixed with 4 ml TBA (0.5%) and the solution was kept in the water bath at the temperature of 95 ºC for half an hour, before being brought to room temperature and centrifuged at 5000×*g* for 10 min at 4 °C. The supernatant was separated from the pellet and its absorbance was checked at 532 and 600 nm. The extinction coefficient for calculating the MDA content is 155 mM^−1^ cm^−1^.

### Estimation of antioxidant enzymes (SOD, CAT, APX)

To estimate antioxidant enzyme SOD, a protocol given by Dhindsa was followed^25^. Cyanobacterial biomass (50 mg) was dissolved in phosphate buffer (2 ml,0.5 M; pH 7.5). The SOD activity of the test sample was assayed by its ability to inhibit the photochemical reduction of Nitro blue tetrazolium. 0.1 ml enzyme extract was mixed with 1.5 ml reaction mixture [1.5 ml Na_2_CO_3_ (1 M), 0.2 ml of methionine (200 mM), 100 μl NBT solution (2.25 mM), 100 μl riboflavin (60 µM), 100 μl EDTA (3 mM) and 1.5 ml phosphate buffer (pH 7.3, 0.1 M)]. For each sample, two sets were prepared. One of these was placed under dark conditions and the another was kept in the light for 10 min. The absorbance of both samples was recorded at λ_560_ against Blank (reaction mixture without enzyme extract). The percentage reductions of samples were calculated and its difference with the blank was estimated, where a single enzyme activity unit refers to the 50% reduction. It was expressed in the unit (enzyme mg protein)^−1^ h^−1^.

CAT activity was determined by evaluating the initial rate of hydrogen peroxide disappearance^26^. Cyanobacterial biomass (50 mg) was homogenized with phosphate buffer (2 ml,0.5 M, pH 7.5) and the mixture was centrifuged at 10,000×*g* for 20 min. The supernatant was separated from the pellet for assay and henceforth referred to as enzyme extract. Enzyme extract (100 μl) was mixed with phosphate buffer (1.6 ml; pH 7.3), 0.2 ml H_2_O_2_ (0.3%), and 100 μl EDTA (3 mM) in a test tube. After 3 min the absorbance of the supernatant was determined at 240 nm against blank. The activity of the CAT enzyme was calculated by using extinction coefficient 0.036 mM^−1^ cm^−1^ and was expressed in nkat mg protein^−1^ (katal = conversion rate of one-mole substrate per second).

The activity of the ascorbate peroxidase enzyme was evaluated by using the catalase extract^27^. The reaction mixture contained phosphate buffer (1 ml,pH 7.3), ascorbate (1 ml; 5 mM), EDTA (100 μl; 3 mM), H_2_O_2_ (200 μl; 0.3%) and enzyme extract (1 ml). The absorbance was checked at 290 nm against a blank (reaction mixture − 0.3% H_2_O_2_). The APX activity was determined with extinction coefficient (2.8 mM cm^−1^) and was expressed in nkat mg protein^−1^.

### Statistical analysis

Results obtained were depicted as mean ± standard deviation (SD). The two-way ANOVA test was applied to compare the effect of different treatments. Two-way ANOVA was selected for this study because it examines the influence of two different independent variables (different treatments and different days) on one continuous dependent variable (IAA excreted).

The significance of the differences among the means was analyzed by Tukey's multiple comparison test (alpha: 0.05, with 5 families and 6 comparisons per family) using GraphPad Prism version- 6.0 (Graph Pad Software, San Diago, CA, USA)". All p-values of < 0.05 are considered significant.

### Molecular docking

Molecular docking of atrazine with D1 protein was performed using the AutoDock-Vina programme (1.1.2). This version performs faster and the calculations are more accurate as compared to AutoDock^28^. The sdf format of the 3D structure of atrazine (CID: 2256) was downloaded from https://pubchem.ncbi.nlm.nih.gov and converted to pdb format using the Avogadro tool. The protein model was prepared using SWISS-MODEL and 1izl.1.Apdb (template). During protein preparation, H_2_O molecules were removed to avoid hindrance, and Kollman charges were added. By using MGL Tools, the PDB file was finally converted to PDBQT^29^.

The docking grid size was set as 66 × 62 × 82 Å with maximum spacing (1 Å) to cover all the residues. Centre of the grid was set at x = 31.163, y = 48.972, z = 45.198. Apart from those mentioned, all other docking parameters were kept at default settings. Modeling results were analyzed using Accelrys Discovery Studio 4.5.

## Results

Cyanobacteria are primary producers which affect many ecological processes including nutrient cycling (e. g. nitrogen fixation) and decomposition in ecosystems. Therefore, adverse effects of atrazine on non-target cyanobacterial species may consequently affect higher organisms^30^. Cyanobacterial reactions to atrazine may depend on various factors including pesticide concentrations, culture conditions, exposure time, and the organism studied^31^.

IAA seems to be an obvious choice of study to counteract the stress in cyanobacteria in crop fields as it is synthesized by cyanobacteria in the presence of l-tryptophan (naturally exuded by crop roots). Some previous research reports have also indicated a link between IAA and stress amelioration in organisms including higher plants^32^.

### Correlation between atrazine and IAA

Before understanding the effect of IAA on stressed cyanobacterium, it is necessary to find out the toxic effect of the atrazine on growth. Therefore, the experiments were divided into two phases:

Effect of atrazine on growth, LC_50_, and specific growth rate.

Effect of IAA on free radical production and antioxidant system of *C. stagnale* in the presence of atrazine.

**Figure 1:**
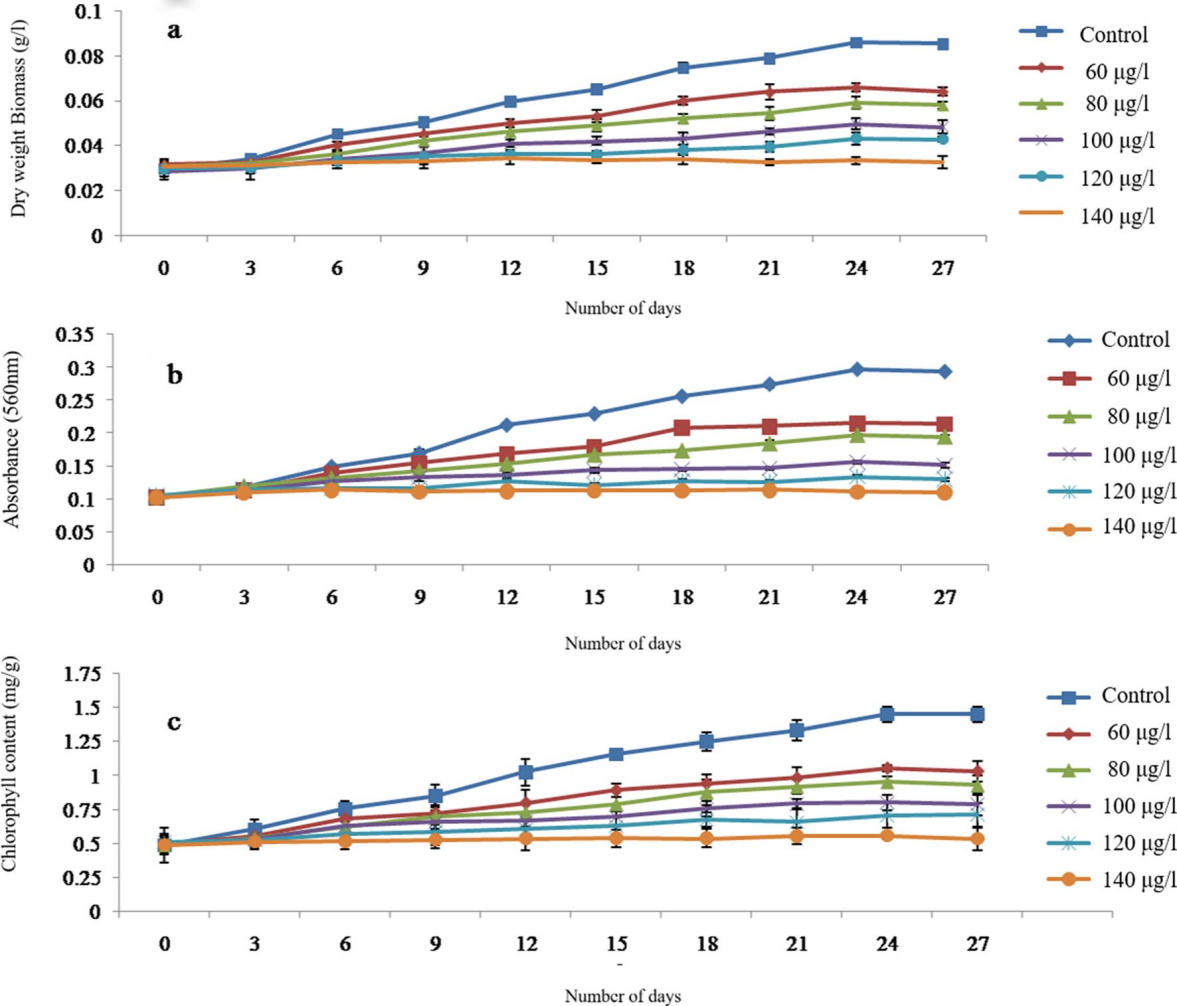
Effect of different concentrations of atrazine on the growth as (**a**) dry weight, (**b**) absorbance and (**c**) chlorophyll of *C. stagnale*. The proceeding table of Two-way ANOVA shows that 3rd day onwards all concentrations of atrazine had a statistically significant effect on the growth of *C. stagnale*. Till the 3rd day of growth, the effect of atrazine on *C. stagnale* was inconspicuous, but as the days progressed it became prominent especially 15th day onwards when the organism entered the log phase of growth. Similar results were found by earlier work on the same strain.

When the *C. stagnale* culture was exposed to multiple doses of atrazine, the growth showed a declining trend in a concentration-dependent manner, measured as dry weight (Fig. 1a), absorbance (Fig. 1b) and chlorophyll content (Fig. 1c). Visibly, the green color of cultures turned yellow/ pale yellow in the presence of atrazine, indicating the stressed state of cyanobacterium.

**Figure 2:**
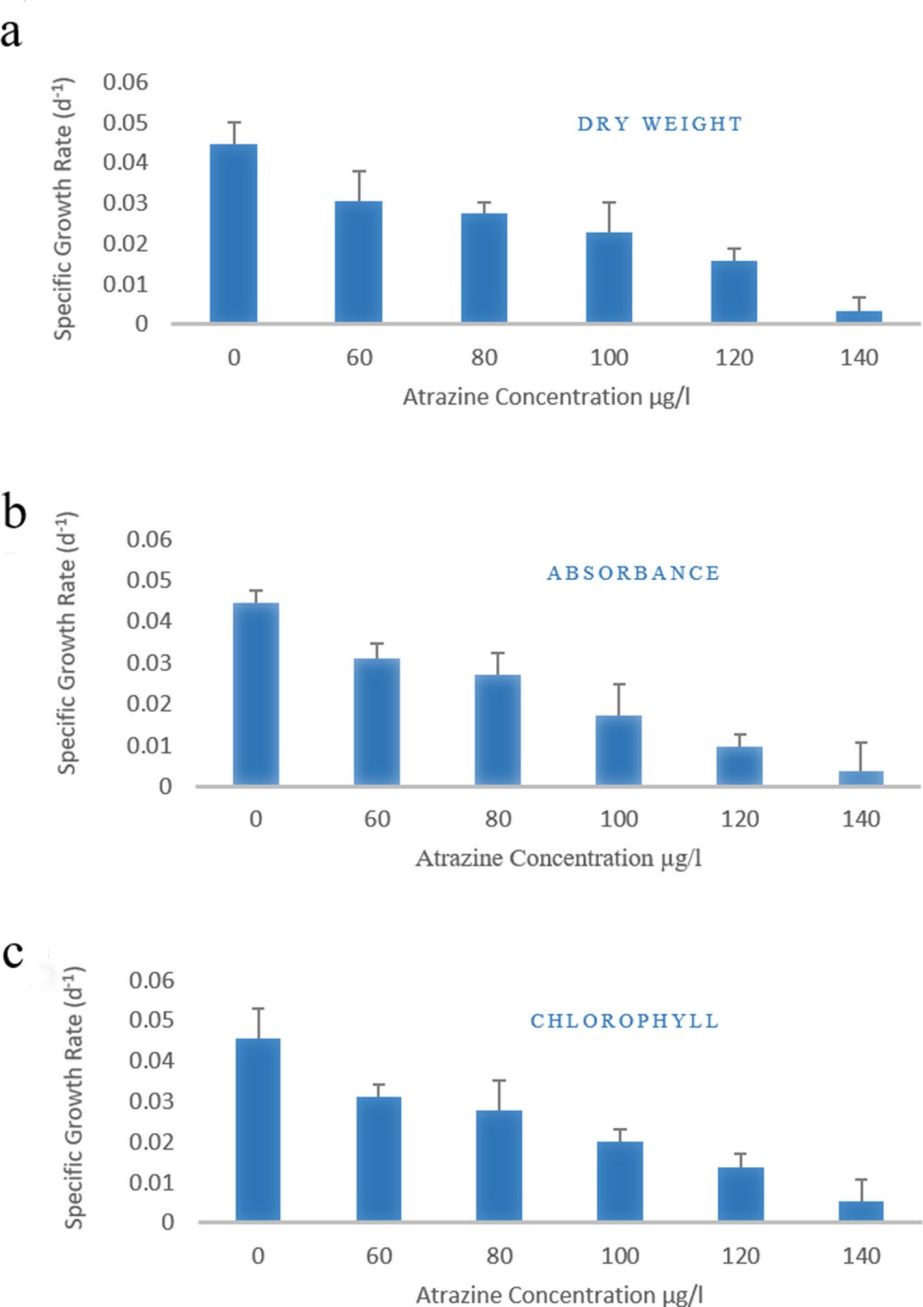
Effect of atrazine on the specific growth rate of *C. stagnale* under atrazine stress.

The atrazine toxicity on the specific growth rate of *C. stagnale* was also calculated by fitting the data (absorbance at 560 nm, biomass values, and chlorophyll) in Eq. (2). The specific growth rate also showed a concentration-dependent decrease with atrazine (Fig. 2).

After finding out the extent of atrazine toxicity on growth, LC_50_ (121.5 µg/l) and specific growth rate, the main target of the present work (role of IAA in amelioration of atrazine toxicity) was studied. This involved a comparative analysis of chlorophyll and protein content, free radical production (as MDA), and antioxidant machinery (SOD, CAT, and APX) of atrazine stressed *C. stagnale* in the presence of exogenous and endogenous IAA.

### Total chlorophyll content

**Figure 3:**
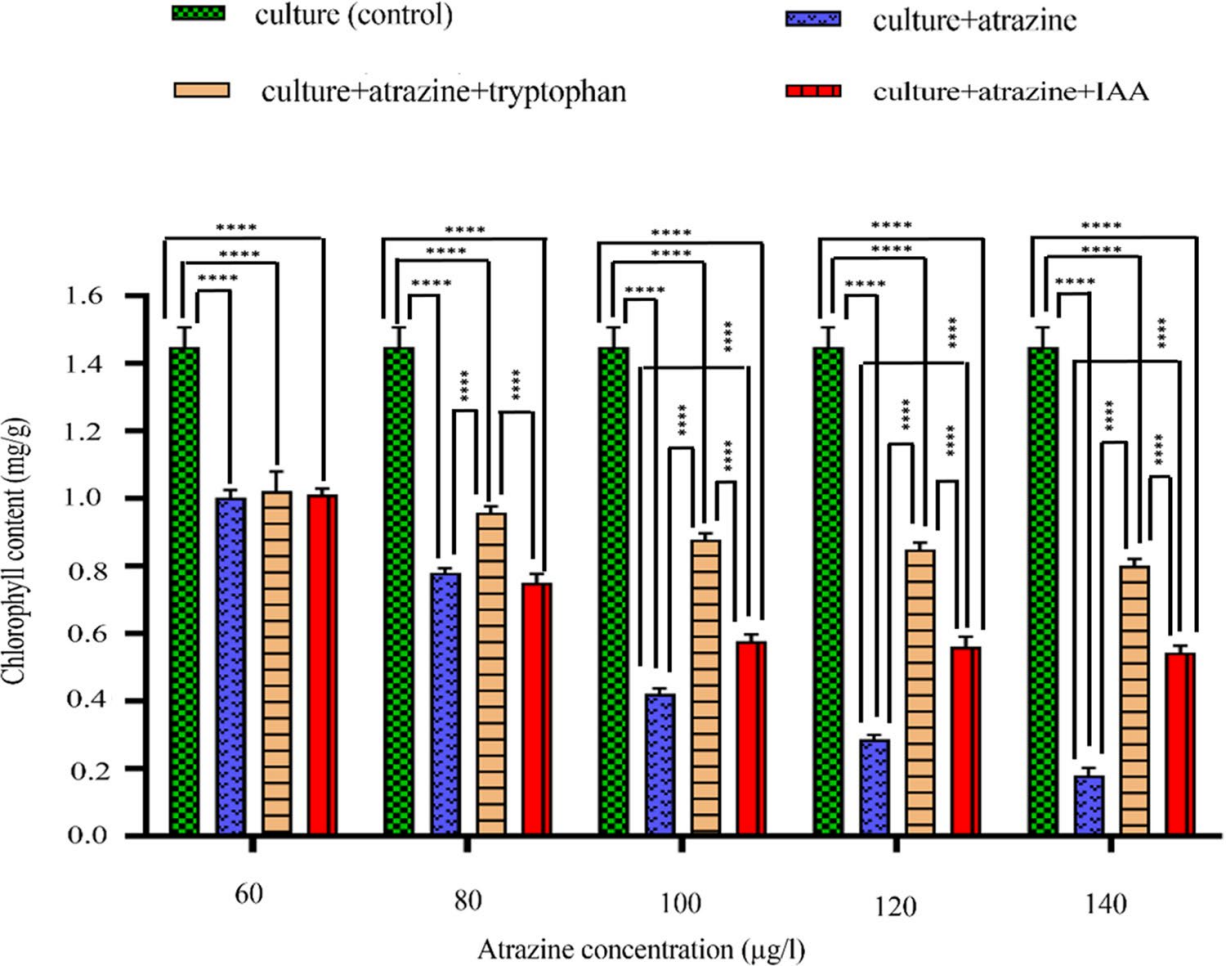
Intergroup and intragroup comparison of mean levels of total chlorophyll content on 24th day in *C. stagnale*, using Two-way ANOVA and Tukey's multiple comparison test respectively (*p < 0.05; **p < 0.01; ***p < 0.001; ****p < 0.0001). The proceeding table for Two-way ANOVA and Tukey's multiple comparison test shows that all the values were statistically significant at all concentrations of atrazine and it can be inferred that all the four treatments had a distinct effect on the total chlorophyll content of the strain across all the studied concentrations of atrazine. The extent of protection was more when IAA was endogenously produced (l-tryptophan dependent) i.e. more chlorophyll was produced when l-tryptophan was provided to the strain in comparison to when IAA was directly provided.

In the presence of only atrazine, the photosynthetic pigment chlorophyll estimated in 24-day old cultures displayed a decreasing trend in a concentration-dependent manner i.e. (9.34%↓, 29.46%↓, 61.86%↓, 74.08495%↓, and 83.78%↓) in comparison to control (Fig. 3). In cultures containing tryptophan and atrazine, the chlorophyll content reduced w.r.t control i.e. 7.68%↓, 13.30%↓, 20.51%↓, 23.20%↓, and 27.69%↓) but increased w.r.t. atrazine stressed cultures. Interestingly, in cultures containing exogenous IAA, the chlorophyll content was even greater than in cultures with tryptophan (endogenous IAA) but was still lesser than the control (8.60%↓, 32.26%↓, 47.76%↓, 0.0001, 49.38%↓, and 50.79%↓). This indicated that the tryptophan-induced IAA synthesis played a more protective role against atrazine toxicity than exogenous IAA.

### Total protein content

**Figure 4:**
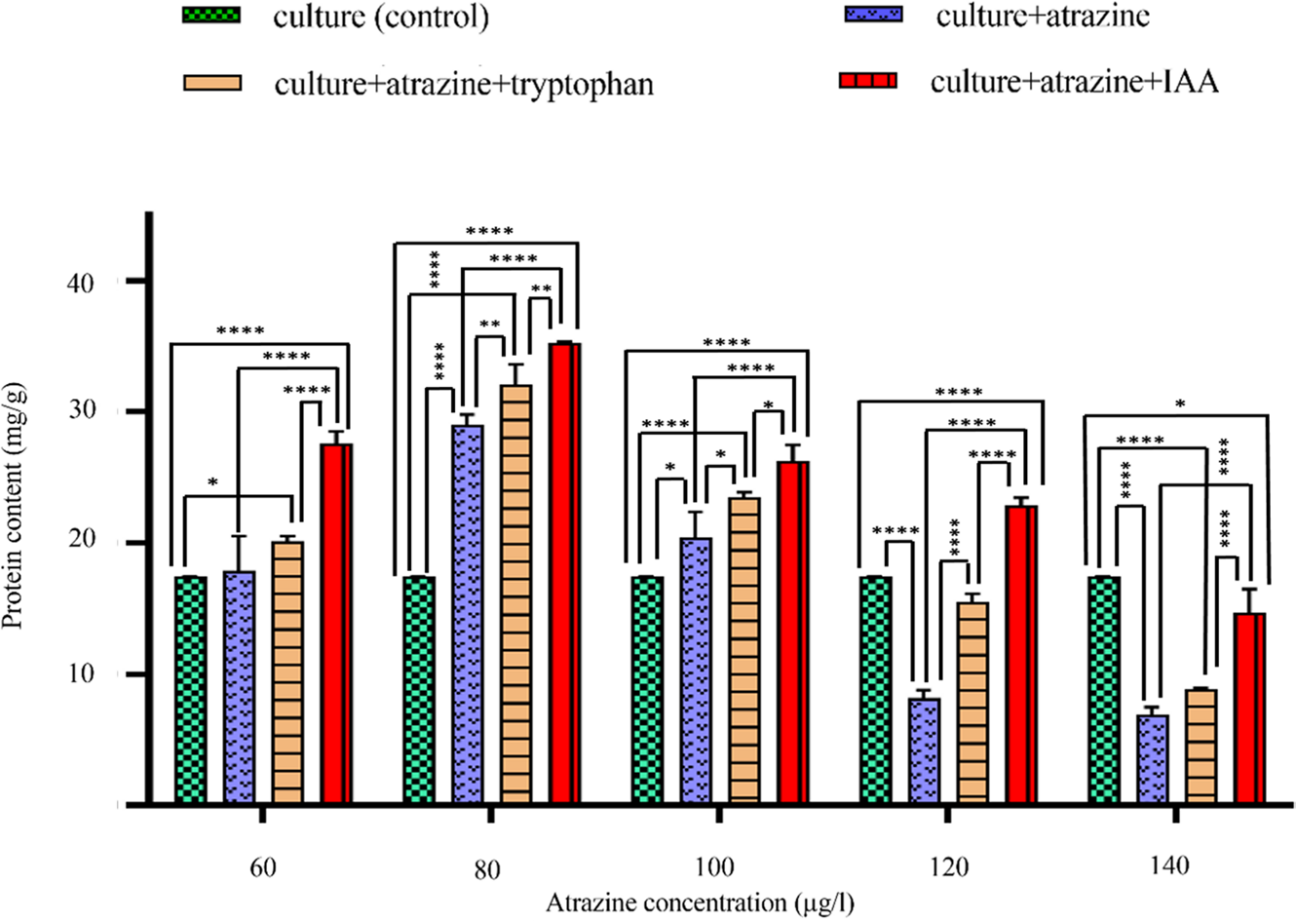
Intergroup and intragroup comparison of mean levels of total protein content on 24th day in *C. stagnale*, using Two-way ANOVA and Tukey's multiple comparison test respectively (*p < 0.05; **p < 0.01; ***p < 0.001; ****p < 0.0001). All the values were found to be statistically significant. Two-way ANOVA analysis and Tukey's multiple comparison test showed that all the values were statistically significant at all concentrations of atrazine and it can be deduced that all the four treatments (control, atrazine, atrazine + l-tryptophan, and atrazine + IAA) had a well-defined effect on the total protein content of the strain through the various concentrations of atrazine.

In the presence of atrazine (60, 80, 100, 120, 140 µg/l) the maximum protein content was observed at 80 µg/l (66.31%↑) (Fig. 4). After supplementation of IAA (both tryptophan-induced endogenous and exogenous), protein content increased till 100 µg/l i.e. 34.72%↑ and 50.66%↑ respectively. Also, cultures containing tryptophan/ IAA contained more protein content in comparison to cultures containing only atrazine at every concentration checked.

### Total MDA content

**Figure 5:**
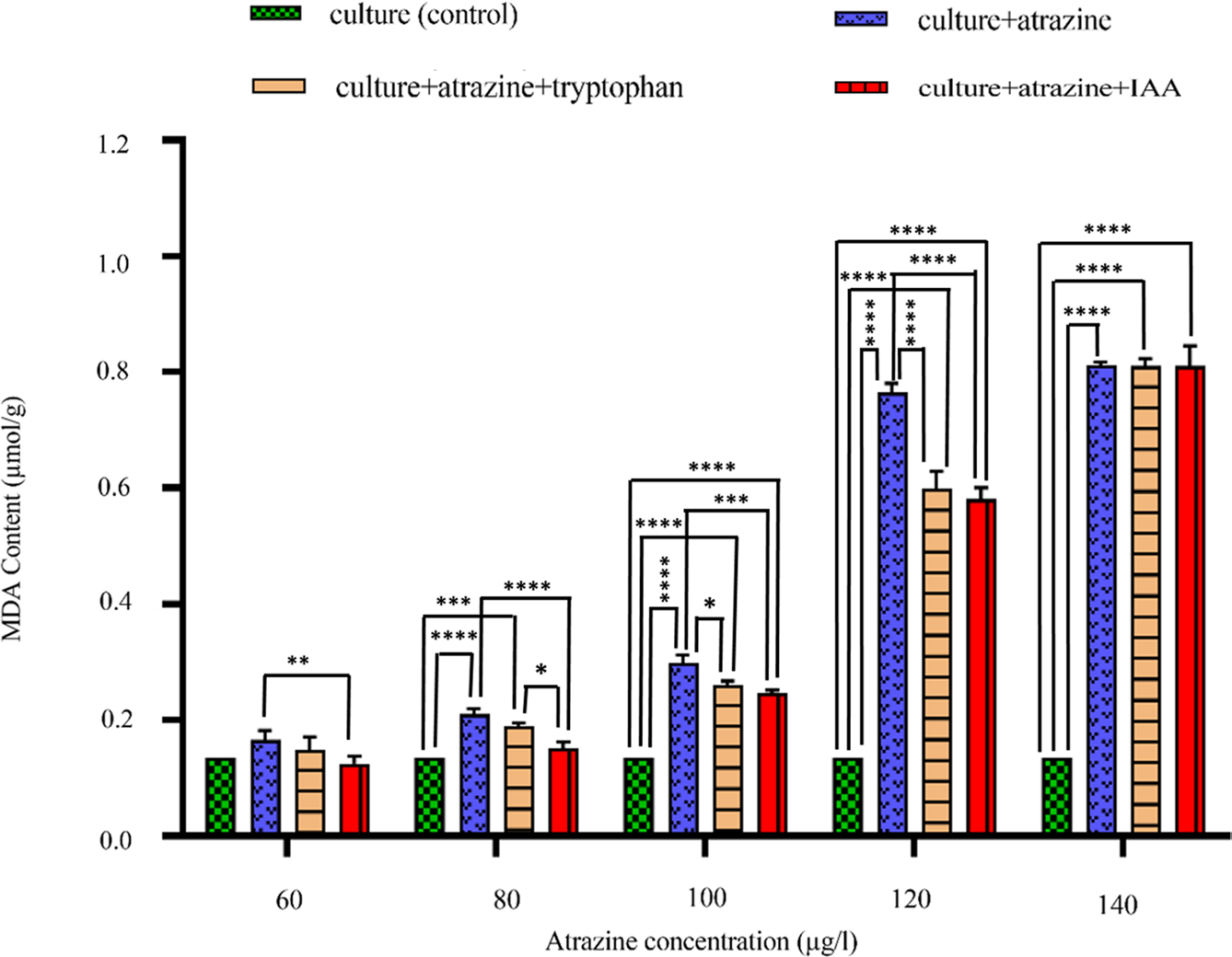
Intergroup and intragroup comparison of mean levels of MDA content on the 24th day in *C. stagnale*, using two-way ANOVA and Tukey's multiple comparison test respectively (*p < 0.05; **p < 0.01; ***p < 0.001; ****p < 0.0001). The ANOVA results for MDA showed that all four treatments had a different effect on the MDA content. Furthermore, Tukey's multiple comparison test demonstrated that at low concentrations of atrazine (60 and 80 μg/ml), all the three treated sets show similar MDA content in comparison to the control, and therefore the difference in the values was statistically non-significant. At higher concentrations of atrazine (100, 120, and 140 μg/ml), the MDA content in all the three treated sets increased drastically, as a result of which the difference was statistically significant with control but the values were similar to each other and therefore the difference was statistically non-significant when the sets containing atrazine were analogized to each other.

At the cellular level, the stress-induced damage is signified by free radical-induced peroxidation of lipid membranes. In cultures exposed to only atrazine (60, 80, 100, 120, 140 µg/l), MDA increased significantly at all studied concentrations in comparison to control (22.85%↑, 114.76%↑, 208.57%↑, 464.28%↑, and 499.04%↑) and the presence of tryptophan/ IAA reduced MDA content at all studied concentrations [(10.47↑, 40.57↑, 93.38↑, 341.90↑, & 498.04↑) and (8.39↓, 11.54↑, 81.63↑, 328.93↑ and 498.04↑)] respectively (Fig. 5).

### Effect of atrazine and IAA on Anti-oxidative enzymes (SOD, CAT, and APX)

The toxic effects of pesticide-induced free radicals are counteracted by escalating activities of their antioxidative enzyme system (such as SOD, CAT, APX, etc.) in photosynthetic organisms. In the current study, the SOD, CAT, and APX activities gradually escalated in a concentration-dependent manner when *C. stagnale* was exposed to atrazine.

**Figure 6:**
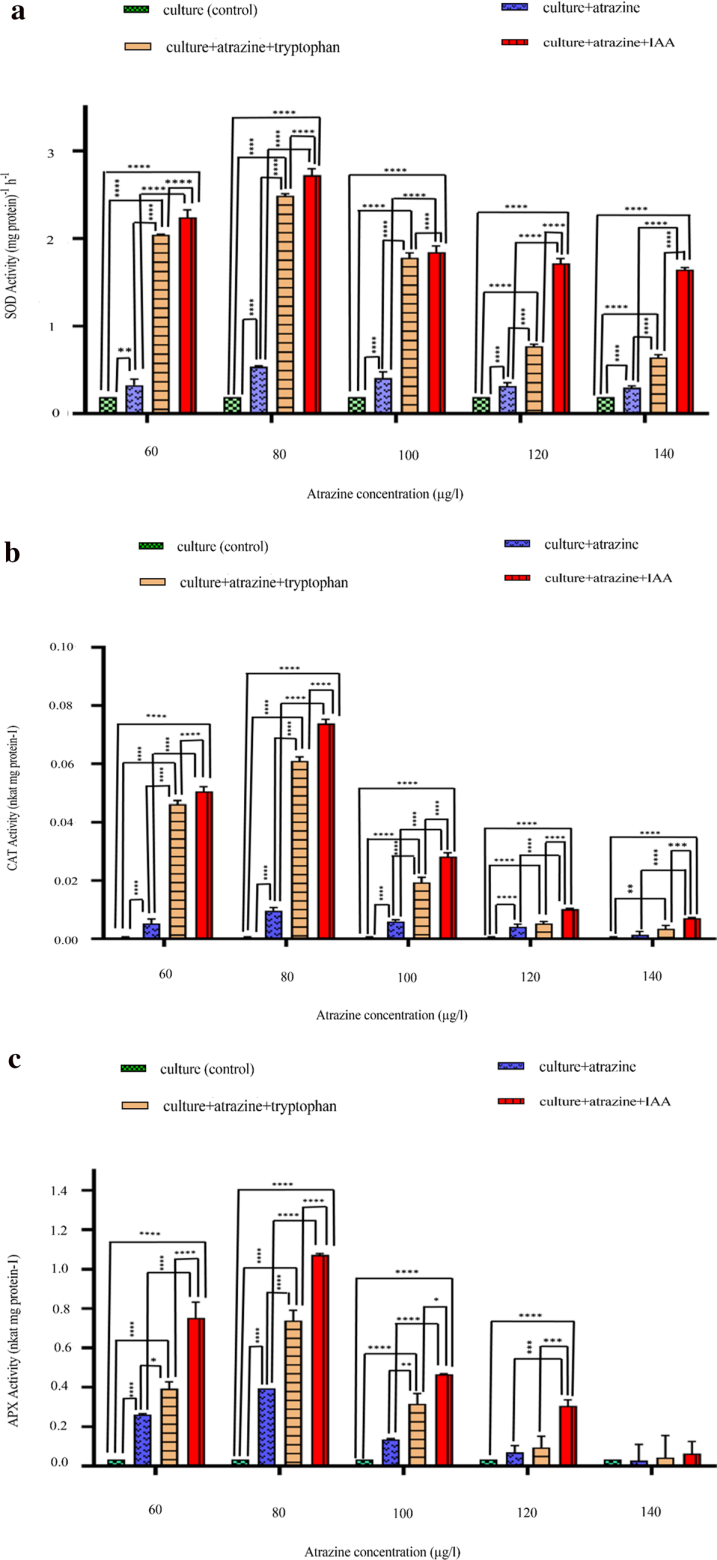
Intergroup and intragroup comparison of mean levels of anti-oxidative enzyme activity (**A** SOD, **B** APX, **C** CAT) on 24th day in *C. stagnale*, using Two-way ANOVA and Tukey's multiple comparison test respectively (*p < 0.05; **p < 0.01; ***p < 0.001; ****p < 0.0001). The proceeding table of Two-way ANOVA shows that all the treatments had a distinct effect on the activities of antioxidative enzymes (SOD, APX, and CAT). Besides, Tukey's multiple comparison test of APX and CAT proved that at lower concentrations of atrazine (60,80, 100 μg/ml) the difference in all the values was statistically significant, which showed that all the treatments had a distinctly different effect at lower concentrations of atrazine on the APX and CAT activities. But at the highest concentration (140 μg/ml), the difference in most of the values was non-significant, showing that at higher concentrations the organism is not able to deal with the stress even in the presence of IAA (endogenous/ exogenous) and the APX and CAT activities were similar in all the sets. According to this test, all the results of SOD were statistically significant, apparently due to the different effects of all the treatments.

Quantitatively, the SOD activities were stimulated significantly with increasing concentrations of atrazine in comparison to control (70.88%↑, 181.36%↑, 113.53%↑, 65.40%↑, and 54.98%↑) (Fig. 6a), which further increased in the presence of IAA (both tryptophan-induced endogenous and exogenous) i.e. [(710.09%↑, 891.30%↑, 572.97%↑, 304.70%↑, and 185.14%↑) and 815.12%↑, 1015.29%↑, 657.11%↑, 380.92%↑, and 238.03%↑) respectively].

CAT activity also displayed an increment in comparison to control (359.40%↑, 565.64%↑, 336.33%↑, 168.42%↑, and 18.92%↑) (Fig. 6b). When IAA (both tryptophan-induced endogenous and exogenous) was supplemented to the atrazine stressed cyanobacterium, the CAT activity increased [708.36%↑, 807.31%↑, 540.56%↑, 358.57%↑, 197.26%↑) and (1204.39%↑, 1579.30%↑, 1222.94%↑, 607.51%↑, and 84.94%↑)] respectively.

Similar to SOD and CAT, APX also showed an increment in its activity in the presence of atrazine at 60, 80,100, and 120 µg/l atrazine but displayed a slight decline in atrazine in comparison to control i.e. {371.20%↑, 616.32%↑, 296.62%↑, 101.68%↑, 12.51%↓} (Fig. 6c). On the addition of IAA (both tryptophan-induced endogenous and exogenous), the APX further increased i.e. [(578.93%↑, 1039.08%↑, 527.65%↑, 173.63%↑ & 24.66%↑) and (1511.99%↑, 2146.93%↑, 1258.83%↑, 794.54%↑ & 83.66%↑) respectively].

### Molecular docking

**Figure 7:**
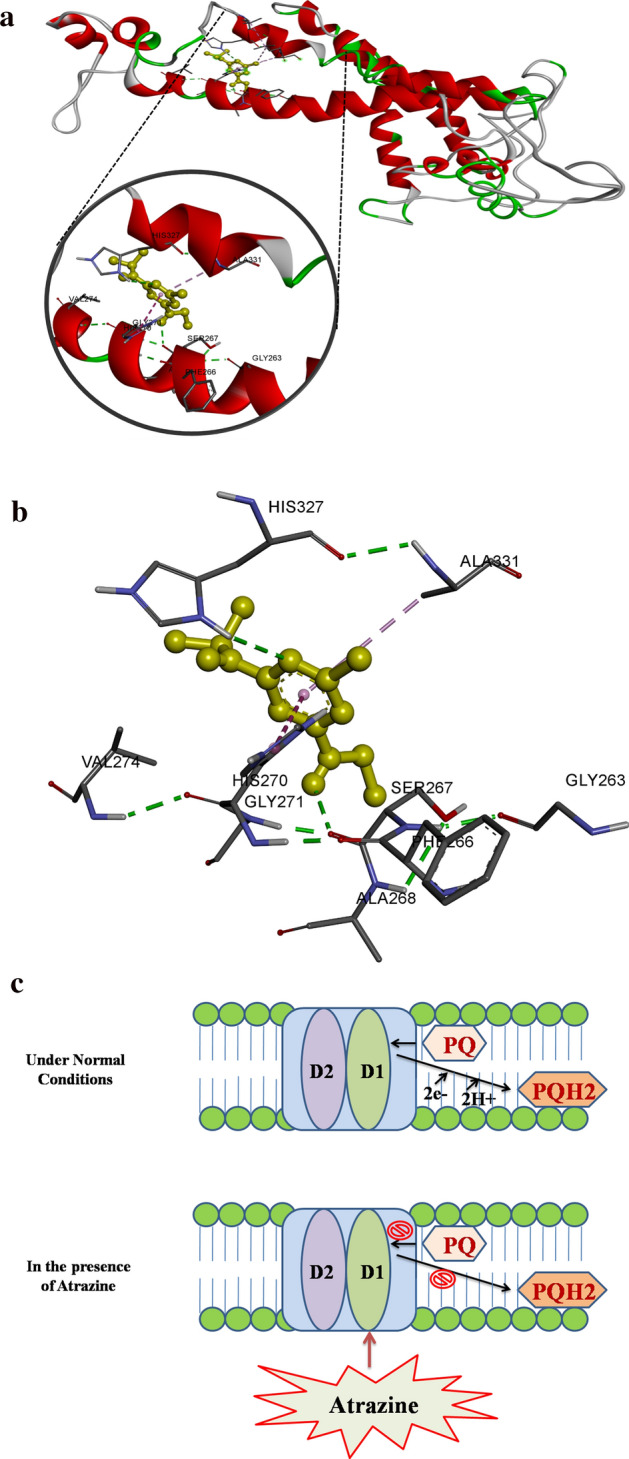
Molecular docking image of Atrazine binding with D1 protein. (**a**) Atrazine is represented as a ball and stick model while D1 protein is shown as a ribbon. (**b**) 2-Dimensional view of various amino acid residues interacting with atrazine through different non-covalent interactions. (**c**) Proposed mechanism of atrazine toxicity in *C. stagnale.*

Molecular docking of atrazine with D1 protein of *C. stagnale* gave nine (9) best conformations with low binding energy, of which the lowest had binding energy of − 4.5 kcal mol^−1^ signifying that this was the most stable complex. In this complex, atrazine was found to interact with several residues of D1 protein and displayed different non-covalent interactions including hydrogen bonds with Ser 267 and His 327 residues, van der Waals interaction with Gly 271, Val 274, His 307, and Leu 330 while His 270 and Ala 331 showed Pi-Pi T-shaped and Pi-Alkyl interactions with atrazine respectively (Fig. 7a,b).

## Discussion

A crucial demand for sustainable agriculture is the endless revitalization of soil with nutrients, reducing the dependence on chemical fertilizers. Administration of diazotrophic cyanobacterium as biofertilizer improves agricultural yield without adversely affecting the environment^33^. Although, pesticides/herbicides are a pre-requisite for modern agricultural practices their application through the ages has given rise to complications due to their toxic effects on soil microflora. Their application leads to the overproduction of ROS that creates oxidative stress in cells damaging various cellular components in the process. Organisms tolerate environmental stresses by synthesizing various enzymatic and non-enzymatic biochemicals that scavenge ROS. Among other compounds, plant growth regulators (IAA, cytokinins, etc.) have been shown to assist organisms in stress amelioration. Considering the potential of plant growth hormones, specifically IAA, the current research work was designed to study the role of IAA in alleviating the toxic effects of atrazine on *C. stagnale*. Initially, the toxicity of atrazine was detected in the cyanobacterial growth and later IAA was checked for any amelioration activity in atrazine stressed organisms.

When grown in an enclosed environment, photosynthetic organisms enter the stationary phase after a few generations due to nutrient limitation and waste accumulation resulting in stunted growth. During the current study, in addition to normal growth studies, the effect of atrazine toxicity was also observed on growth and specific growth rate as absorbance at 560 nm, dry weight biomass, and total chlorophyll content for 27 days. Atrazine reduced the growth in a concentration-dependent manner resulting in a reduced specific growth rate.

Atrazine affects growth in an organism by inhibiting the electron transport chain during photosynthesis in turn diminishing the normal cell division subsequently leading to several secondary effects, including modification in the synthesis of proteins, amino acids, and nucleic acids^34–36^. After finding out the toxicity pattern of atrazine on *C. stagnale* subsequent experiments were performed to understand the effects of IAA/ tryptophan on atrazine stressed in *C. stagnale*.

In *C. stagnale* the photosynthetic pigment (chlorophyll) showed an overall decreasing trend till 140 µg/l of atrazine. The reduction was more pronounced at higher atrazine concentrations. Atrazine has been shown to decrease the chlorophyll content in different cyanobacterial sp. like *Oscillatoria limnetica, Arthrospira* sp., *Synechococcus* sp., and *Anabaena variabilis*^37^. Reduction of photosynthetic pigment content in cyanobacteria by other pesticide treatments has also been reported e.g. endosulfan resulted in chlorophyll reduction in *Aulosira fertilissma, A. varaibilis*, and *Nostoc muscorum*^38^. Many mechanisms have been suggested for chlorophyll reduction under pesticide stress. They either interfere with chlorophyll production by hampering the synthesis of porphyrin rings^39^or increase its degradation by enhancing the ROS production^40^. Like other pesticides/ herbicides, atrazine affects the algal cell by changing pigment profile and photosynthetic capacity^41^.

During the present work, IAA (both L-tryptophan induced endogenous and exogenous) reduced the deleterious effects of atrazine that resulted in improved growth (as chlorophyll content). Further, it was found that chlorophyll content was higher in the presence of L-tryptophan supplemented endogenous IAA signifying a more effective role in combating the atrazine stress than the exogenous IAA. In previous studies also, IAA supplementation has shown to increase chlorophyll content in *Zea mays*^20^, wheat^15^, *C. vulgaris*^42^, and *Balanites aegyptiaca*^43^ under salt, cadmium stress and normal conditions respectively. In a study on IAA producing *Leclercia adecarboxylata,* its induction leads to the increased chlorophyll fluorescence in *Solanum lycopersicum* L, under normal and stress conditions signifying the positive effect of microbial IAA on stressed plants^44^.

The total protein content (per mg dry algal biomass) of the cell under all three studied conditions in *C. stagnale* significantly increased till 80 µg/l and then showed a gradual decline. It may be due to increased (non-specific) protease activity along with reduced carbon assimilation^45^. Previously, similar observations have been reported in *N. muscorum* and *A. variabilis* with herbicides butachlor and thiobencarb^46,47^ *A. fertilissma*, *A. varaibilis*, and *N. muscorum* under endosulfan stress^38^ and *Spirulina platensis* under heavy metal (cobalt) toxicity^48^. The increase in protein at low concentrations may be due to the synthesis of stress retarding proteins. Under stress, organisms initiate gene expression and synthesis of stress (induced) proteins that help to tolerate adverse conditions^49^. Cyanobacteria synthesize multiple proteins when faced with stressful conditions^50^. Such stress proteins have previously been studied under heat shock^51^, desiccation^52^, nitrogen deficiency^53^, and salt stress^54^.

At every studied atrazine concentration, the presence of IAA (both extracellular and intracellular) counteracted the toxic effect by increasing protein content in a cyanobacterium. This effect was more pronounced at 80 µg/l atrazine. Cultures containing exogenous IAA were found to be more effective i.e. produced more protein content in comparison to endogenous IAA (L-tryptophan). During previous studies also, IAA leads to the induction of significant protein content assimilation under normal conditions in *Chlorella vulgaris*^55^, salt stress in Sorghum^56^, and *Hibiscus sabdariffa*^57^.

Oxidative stress in *C. stagnale* by atrazine was confirmed by measuring the amount of MDA generated. In all studied sets, the MDA content demonstrated a concentration-dependent increase with atrazine; indicating lipid peroxidation and induction of oxidative damage. Providing IAA (exogenous and endogenous) to *C. stagnale* decreased MDA content at low concentrations of atrazine, revealing a role of IAA in the detoxification of free radicals. But at its higher concentration of atrazine (i.e. 140 µg/l), IAA could not help the organism in counteracting the adverse effects. During previous studies also, pesticides like endosulfan and bentazon showed MDA production in *N muscorum, A. varaibilis, A. fertilissma*, and *Anabaena cylindrical*^38,58^.

Reactive oxygen species (ROS) are extremely reactive and therefore highly toxic species that can oxidize multiple cellular components like DNA, RNA, and lipids resulting in membrane damage and lipid peroxidation, ultimately leading to cell death^59^. In cells, ROS remain under tight regulation during normal conditions, therefore all ROS generated are readily converted to harmless forms. But under stress conditions, the rate of ROS generation exceeds the rate of its conversion leading to various adverse effects. To scavenge these reactive species, cells produce and express several antioxidant enzymes^38^. For example, under endosulfan stress, *Plectonema boryanum, A. fertilissma, A. variabilis,* and *N. muscorum* produce SOD, CAT, and APX enzymes^38,40^. SOD functions as an antioxidant enzyme by transforming the superoxide radical (O_2_^·-^) to molecular oxygen (O_2_) and H_2_O_2_^60^whereas CAT and APX facilitate the removal of H2O2 from the system.

In the present study, activities of all studied anti-oxidative stress enzymes (SOD, CAT, and APX) showed a considerable enhancement in comparison to control till 140 µg/l atrazine, but the highest antioxidant enzyme activities were found at 80 µg/l concentration, implying that the organism had problems tolerating atrazine beyond this concentration. Enhanced activity of APX in comparison to CAT signified that APX played a greater role in H_2_O_2_ sequestering by *C. stagnale* under atrazine stress. The pattern of SOD, CAT, and APX response to increased concentrations of atrazine was very similar. These enzymes act cooperatively to ameliorate oxidative stress^61^.

IAA negated the harmful effects of atrazine as enzyme activities enhanced in the presence of IAA and were higher exogenous IAA was applied and atrazine when paralleled to endogenous IAA (as L-tryptophan). It is suggested that IAA enhances the antioxidant potential by changing the stress-responsive genes expression^14^. IAA has been shown to alleviate toxic effects of lead and zinc toxicity in *H. annuus* L^17^, cadmium toxicity *T. aestivum*^15^, oxidative, cold shock, heat shock, desiccation and osmotic stress in *B. japonicum*^16^, salt stress in *P. mungo* L. and *Solanum melongena* L.^62^ *T. aestivum*^19^ and furadan (pesticide) toxicity in *Nostoc* sp. and *Anabaena* sp.^18^. It also effectively alleviated drought-induced oxidative damage via modulation of antioxidant enzymes and peroxidase activity in *Trifolium repens* and *T. aestivum* respectively^63,64^.

During the current research work, the interaction of atrazine with the D1 protein of *C. stagnale* was studied with the help of molecular docking to understand whether an interaction of atrazine with the studied cyanobacterium causes any changes at molecular level. The non-covalent interactions of atrazine with different amino acid residues (Ser 267, His 327, Gly 271, Val 274, His 307, Leu 330, His 270, and Ala 331) of D1 protein caused notable hindrance of PS-II catalyzed electron transport due to damage of D1 protein and alterations in water oxidation complex. It results in the displacement of plastoquinone (QB) from its binding site in the D1 protein photosystem II (PS II), blocking the conversion of plastoquinone to PQH2 (Fig. 7c)^65^. Such inhibition of photosynthesis results in an oxidative burst in a cell which harms lipids and membrane integrity and the resulting oxidative damage may lead to cell death^66^. Hence, the results indicated that the interaction with atrazine lethally affected the cyanobacterium.

IAA counteracts the destructing effects of atrazine on organisms by facilitating the de novo synthesis of D1 protein during the PSII repair cycle, thereby, negating the toxic effect of atrazine. Such positive effects of IAA supplementation on stressed organisms have also been previously observed^15^.

## Conclusion

Present work demonstrated that the studied cyanobacterium *C. stagnale* possesses the highest IAA (growth-promoting hormone) production capacity whose growth decreased with increasing concentrations of atrazine through free radical (measured as MDA). IAA supplementation counteracted the adverse effects of the pesticide and revitalized growth by increasing biomass, chlorophyll, and protein content and activities of antioxidant enzymes (CAT, SOD, and APX). IAA was more effective when provided exogenously than endogenously (after l-tryptophan induction) due to its easy accessibility for direct use.

## Supplementary Information

Supplementary Figures.
