# Supplementary material for: Ameliorative effects of endogenous and exogenous indole-3-acetic acid on atrazine stressed paddy field cyanobacterial biofertilizer Cylindrospermum stagnale
Source: Sci Rep. 2022 Jul 1;12:11175. doi: 10.1038/s41598-022-15415-z (PMC9249835; doi:10.1038/s41598-022-15415-z)

Fig. S1: LC_{50} of atrazine in *C. stagnale* under atrazine stress

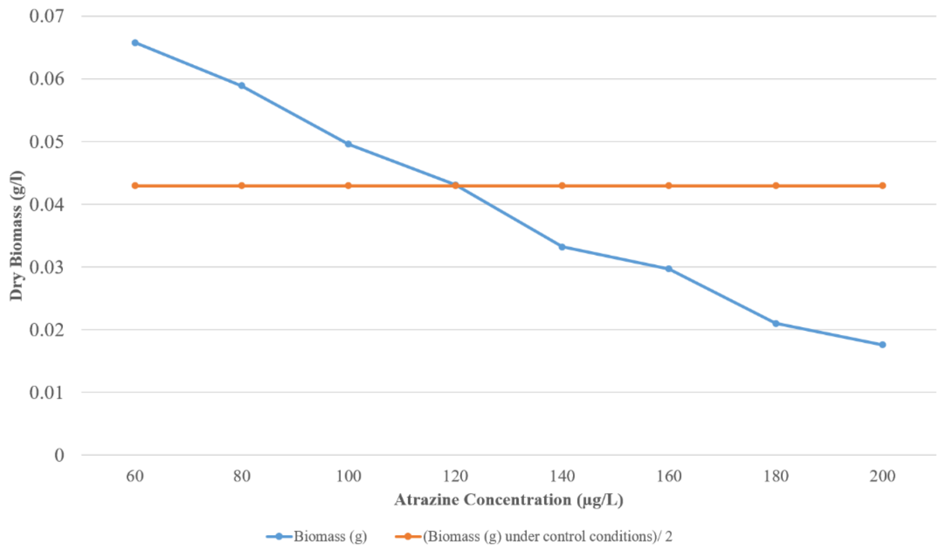

Fig. S2: Growth curve of *C. stagnale* in atrazine and IAA solvents.

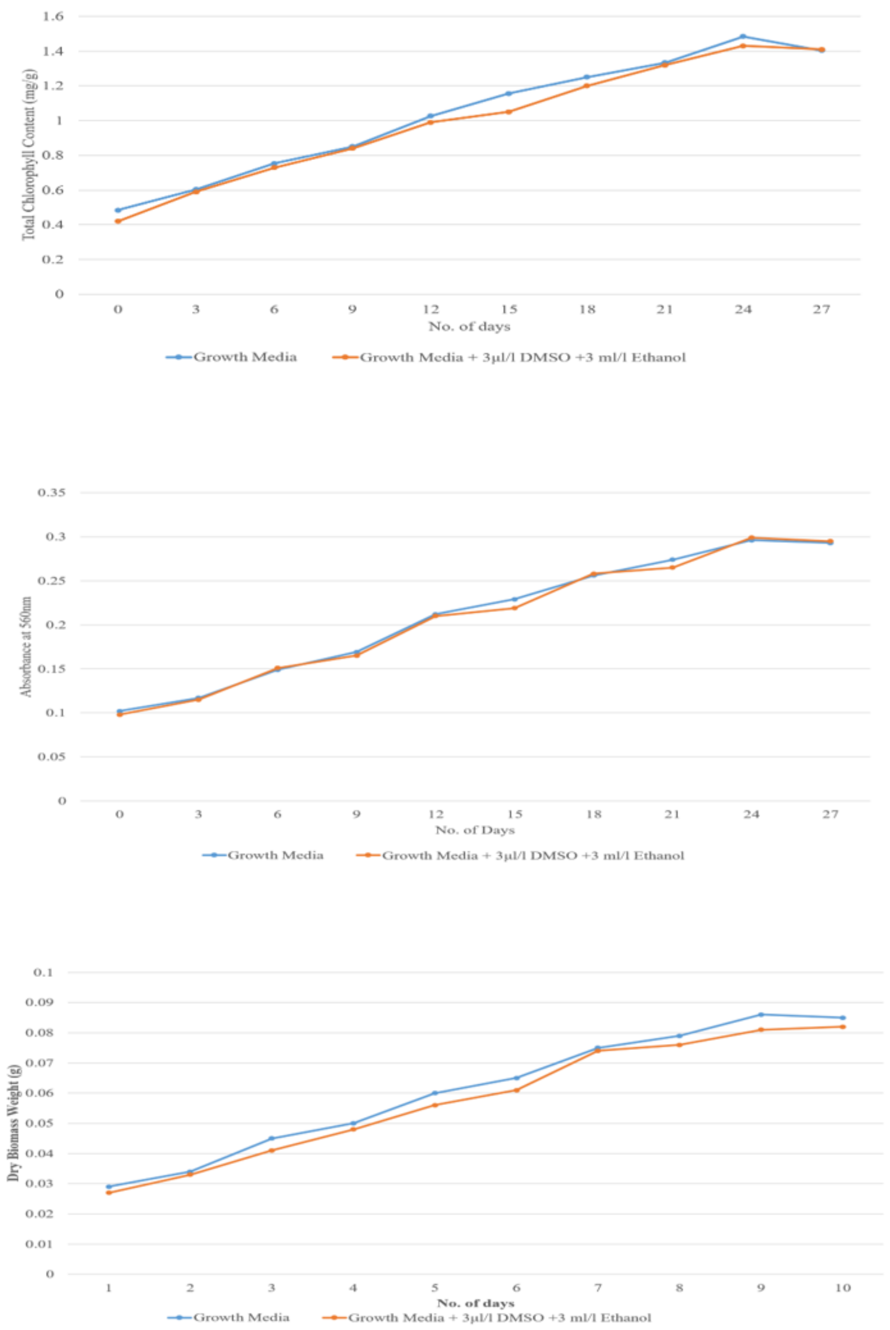

Supplement: Supplementary file 1 — Supplementary Figures. [file 41598_2022_15415_MOESM1_ESM.pdf]
